# Editorial: Post-feminist practices, subjectivities and intimacies in global context

**DOI:** 10.3389/fsoc.2023.1153965

**Published:** 2023-02-22

**Authors:** Mehita Iqani, Caio Simões de Araújo

**Affiliations:** ^1^Journalism Department, Stellenbosch University, Stellenbosch, South Africa; ^2^Wits Institute for Social and Economic Research, University of the Witwatersrand, Johannesburg, South Africa

**Keywords:** post-feminism, intimacy, gender, sexuality, global flows, Global South

In recent decades, “post-feminism” has become a recurrent theme and a buzzword of sorts in a variety of settings, from academia and politics to media discourses and popular culture. After decades of scholarship and public debate on the matter, the task of delimiting the boundaries of what “post-feminism” means seems intellectually futile, particularly because the ambiguity of the term is what makes it intriguing and compelling as a frame of analysis.

In a particularly relevant interpretation, scholars of gender have taken post-feminism as promising conceptual lenses with which to interrogate the (un)doing of gendered subjectivities in our contemporary culture, where intersectional inequalities are (re)produced amidst the neoliberal celebration of individual agency and empowerment, as predicated on consumption and commodification of gender difference. Postfeminism extends understandings of the ways in which gender is performed, and highlights how neoliberal capitalism and market values intersect with identity, subjectivity and agency (Gill and Scharff, 2013).

This Research Topic intervenes in this ongoing debate by focusing on the practices, subjectivities and intimacies that are shaped by post-feminist cultural and political formations, whatever these may mean across the globe, and in diverse social, cultural, and political contexts. A significant body of scholarship interrogating the notion of post-feminism from the standpoint of the Global North already exists, even if they have been critiqued for their narrow focus on white middle class subjectivities (Butler, 2013). As important as those debates are, we were specifically interested in research that critically engages with the complexities of post-feminism from Global South perspectives, a work that is already being done (Dosekun, 2015). This collection of research articles succeeds in bringing together voices and arguments from Africa, Asia and Eastern Europe: three regions where new attitudes to the complex intersection of gender with race, class, caste, religion, and more are forging new lived experiences (and therefore theoretical positions) on identity and subjectivity. As such it serves as an intervention combining elements of cultural studies, feminist critique and media analysis with methods and analytical frames from the social sciences. The collection centers transnational research in and around the Global South and offers a platform for extending our understandings of post-feminisms beyond existing western frameworks.

From this collaborative conversation, one key theme that has emerged is a remixing of the familiar argument of gender as performed and performative, and gender as fluid: with post-feminism being a useful theoretical tool for exploring how gender intersects with neoliberal power and material culture in specific ways. Performed gender relates directly to the intimate, the bodily, to relationships and senses of self, and how these are shaped by tangible practices embedded in everyday life, theorized as “self-fashioning” (Dosekun, 2020; Roy, 2022). Relating to these insights, a first set of articles examines practices of self-fashioning and claim-making in the context of diasporic communities in the UK, feminist activists in Eastern Europe, and bisexual women in South Africa.

Dutta looks at the aesthetic labor of bikini waxing, as practiced by South Asian women in London. Her ethnography presents the complexities of intimacy and disgust attendant to such personal body practices.

Myzelev examines the feminist politics of crafting and handwork, and argues that it can function as “a language of political and social struggle” in Ukraine and Belarus. Through “craftivism,” women are able to articulate both a feminist and nationalist politics.

Khuzwayo writes about the politics of coming out for bisexual women. Divergent views on the necessity and personal value of coming our shows that some realms of sexuality remain in opaque and difficult-to-theorize realms of intimacy.

In a second set of articles, authors analyse how postfeminist formations shape practices, subjectivities and intimacies in the Global South, especially in the context of longstanding intersectional patterns of exclusion (including class, race, and caste) and in light of the neoliberal emphasis on inclusion through consumerism (Lazar, 2009). It is apt that the collection emphasizes the agentic subject—as an object of analysis alongside, and not separated from, the more structural dimensions of discourse, representation, transnational media culture and neoliberal governmentality (Tasker and Negra, 2007). Postfeminist sensibilities emerge through the politics of intimacy and gender-making in the everyday and prosaic moments of lived experience. The articles are invested in examining various cultural forms and media products, from music to social media and TV shows, by which postfeminist sensibilities and gendered imaginaries are mediated and circulated to broad publics.

Rens analyses popular Afrobeats music videos to parse the heteronormative dynamics of masculine and feminist characters. In the romances visually depicted alongside and in the songs, he argues that an ethic of “misogyrom” is in operation, which undermines the agency of the women characters in the videos.

Dunn and Falkof explore how young women who post self-portraits on social media work to create a sense of authenticity, which operates at both the level of feeling and appearance.

Ghosh analyses television shows about Indian matchmaking, and teases out the web of cultural politics regarding nationhood, conservatives, caste and class, while showing how postfeminist values shape the narratives.

Boshoff and Mlangeni examine South African tabloid newspaper writings about older well-off women who have romantic relationships with younger men. The authors show that though the women seem putatively empowered, their agency women should be placed in historical and global context.

The third set of articles examines how the current wave of right-wing politics and anti-feminist backlash sweeping the world poses critical and specific questions to post-feminist dynamics and imaginings in global south contexts. As scholars have showed elsewhere, the contemporary political climate has invited sexist revivals and reactionary understandings of gender and gender theory (Whelehan, 2000; Corredor, 2019; Graff and Korolczuk, 2022). The articles in this issue address some of these questions from the point of view of NGO-led programmes and emerging digital cultures in India and Indonesia.

Chakraborty writes about the “interstitial intimacies” that emerge in labor in an NGO's community-based program to prevent violence against women and girls in Mumbai's urban poor neighborhoods.

Maryani et al. discuss the rise of anti-feminist discourse on social media in Indonesia. This is put into context of the religious and political complexities of Indonesian society.

Through these 10 articles, this Research Topic collection put the spotlight on transnational thinking about postfeminism, revealed the cracks, complexities, and contestations that characterize debates about the economic empowerment of women in contexts of inequality. This collection also gestures toward new directions in the critical analysis of postfeminism, and theory building from the south. It has given full attention to the ways in which postfeminist practices, identities and intimacies intersect with race, ethnicity, indigeneity, and caste, including practices of resistance to oppression organized along such lines. Future work must continue to develop fuller understandings of how neoliberal femininities explicitly matter in relation to racial identities and racialized experiences of life.

This special issue proposes that, in the global south, postfeminism demands an expansive and inclusive understanding of the feminine to include all femininities, in order to explore what the pre-fix “post” means in relation to this inherent diversity. While the articles have tackled these questions from various perspectives and locations, more work is required to integrate trans-femininities into the work of the study of global south postfeminism, as part of a broader politics of rejecting cis-feminist exceptionalism.

## Author contributions

Both authors listed have made a substantial, direct, and intellectual contribution to the work and approved it for publication.
